# β-cell Smad2 null mice have improved β-cell function and are protected from diet-induced hyperglycemia

**DOI:** 10.1016/j.jbc.2021.101235

**Published:** 2021-09-25

**Authors:** Mohamed Saleh, Nada A. Mohamed, Anuradha Sehrawat, Ting Zhang, Madison Thomas, Yan Wang, Ranjeet Kalsi, Justin Molitoris, Krishna Prasadan, George K. Gittes

**Affiliations:** 1 Division of Pediatric Surgery, UPMC Children's Hospital of Pittsburgh, Pittsburgh, Pennsylvania, USA; 2 Division of Pediatric Endocrinology, UPMC Children's Hospital of Pittsburgh, Pittsburgh, Pennsylvania, USA

**Keywords:** SMAD transcription factor, β-cell, glucose metabolism, insulin secretion, transforming growth factor beta (TGF-β), BrdU, bromodeoxyuridine, ER, endoplasmic reticulum, GSIS, glucose-stimulated insulin secretion, HbA1c, glycated hemoglobin, HFD, high-fat diet, HOMA-IR, homeostatic model assessment–estimated insulin resistance, IHC, immunohistochemistry, IPITT, intraperitoneal insulin tolerance testing, PPX, partial pancreatectomy, RIP, rat insulin promoter, RT-PCR, real-time PCR, smad2-βKO, deletion of smad2 protein in *ins1*^*cre*^*;smad2*^*fx/fx*^, T2DM, type 2 diabetes mellitus, TGF-β, transforming growth factor-beta

## Abstract

Understanding signaling pathways that regulate pancreatic β-cell function to produce, store, and release insulin, as well as pathways that control β-cell proliferation, is vital to find new treatments for diabetes mellitus. Transforming growth factor-beta (TGF-β) signaling is involved in a broad range of β-cell functions. The canonical TGF-β signaling pathway functions through intracellular smads, including smad2 and smad3, to regulate cell development, proliferation, differentiation, and function in many organs. Here, we demonstrate the role of TGF-β/smad2 signaling in regulating mature β-cell proliferation and function using β-cell-specific smad2 null mutant mice. β-cell-specific smad2-deficient mice exhibited improved glucose clearance as demonstrated by glucose tolerance testing, enhanced *in vivo* and *ex vivo* glucose-stimulated insulin secretion, and increased β-cell mass and proliferation. Furthermore, when these mice were fed a high-fat diet to induce hyperglycemia, they again showed improved glucose tolerance, insulin secretion, and insulin sensitivity. In addition, *ex vivo* analysis of smad2-deficient islets showed that they displayed increased glucose-stimulated insulin secretion and upregulation of genes involved in insulin synthesis and insulin secretion. Thus, we conclude that smad2 could represent an attractive therapeutic target for type 2 diabetes mellitus.

Diabetes is a significant health problem in the United States and worldwide. According to the National Diabetes Statistics Report by the Centers for Disease Control and Prevention, the prevalence of diabetes in the United States in 2015 was 30.3 million (9.4%), and 84.1 million Americans had prediabetes. Diabetes remains the seventh leading cause of death in the United States (1). With the worsening obesity epidemic, the incidence of type 2 diabetes mellitus (T2DM) has been rising (2). Long-standing obesity and insulin resistance place significant stress on the pancreatic β-cell, causing β-cell dysfunction and progressive β-cell loss, eventually leading to overt diabetes (3, 4). Studying β-cell biology and specifically signaling pathways that regulate insulin secretion, β-cell proliferation and the adaptive capacity of β-cells are all crucial for developing new therapeutic strategies for T2DM. The transforming growth factor-beta (TGF-β) family is a prominent extracellular signaling pathway that regulates essential functions of mammalian cells, specifically proliferation, differentiation, morphogenesis, metabolism, and cell death (5). In general, TGF-β signaling tends to both limit epithelial proliferation and activate cell death processes to maintain homeostasis in mature tissue (5, 6).

TGF-β/smad signaling involves binding of TGF-β superfamily ligands (*e.g.*, TGF-βs 1, 2, and 3, activins, and inhibins) to transmembrane receptors that activate receptor-regulated intracellular smads (R-smads, including smad2 and smad3) (7). The activated R-smads form a complex with the common smad (smad4) and translocate to the nucleus to regulate the transcription of target genes (8, 9). In the pancreas, TGF-β/smad2 signaling has been implicated in the regulation of endocrine maturation and development, where smad2 inhibition during pancreas development leads to increased proliferation of double-hormone–positive immature endocrine cells (10). In the mature pancreas, several studies have shown that suppression of TGF-β signaling increases β-cell proliferation, both in mice (11, 12, 13) and in human islets transplanted into *NOD-scid IL2Rg*^*null*^ mice (14). In addition to a regulatory role in proliferation, other studies have shown that insulin expression and release are negatively regulated by TGF-β signaling (15, 16, 17). In the present study, we used β-cell-specific deletion of smad2 expression to investigate the role of smad2 in β-cell function and proliferation, both at baseline conditions and after a nondiabetogenic loss of β-cells (partial pancreatectomy [PPX]), as well as in a high-fat diet (HFD)-induced hyperglycemia model.

## Results

### Generation of β-cell-specific smad2 null mice

**Figure 1:**
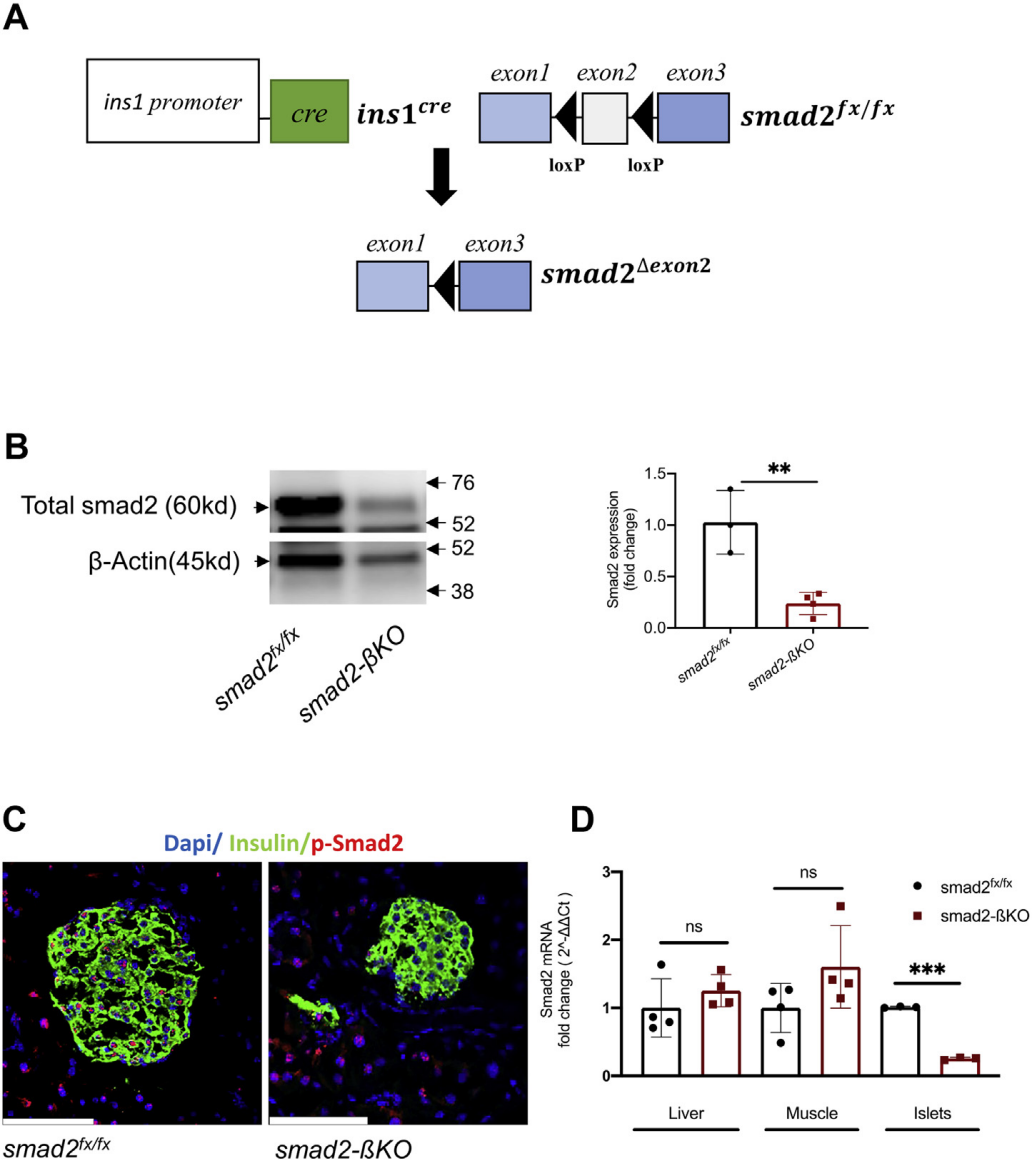
**Generation of β-cell-specific smad2 null mice.***A*, schematic for *ins1*^*cre*^ mediated deletion of smad2. The *Smad2*^*fx/fx*^ mice possess loxP sites flanking exon2 of the genomic smad2. By crossing *smad2*^*fx/fx*^ mice with *ins1*^*cre*^ transgenic mice, we generated β-cell-specific smad2 null mutant (smad2-βKO) mice. *B*, the Smad2 protein expression was downregulated in isolated islets from the 14-week-old smad2-βKO mice compared with the littermate controls. Cropped gels are displayed (*left panel*). Western blot results were quantified by densitometry (*right panel*). β-Actin was used as a protein loading control for western analysis. *C*, representative pancreatic tissue sections from the 14-week-old smad2^fx/fx^ and smad2-βKO mice (n = 3 per group) were immunostained for p-smad2 (Ser-465/467), showing decreased detectable p-smad2 in smad2-βKO mice. *D*, Smad2 mRNA measured by real-time PCR from the liver, muscle, and pancreatic islets of the 14-week-old *smad2*^*fx/fx*^ (*black circles*) and smad2-βKO (*red squares*) mice; n = 3 to 4 per group. Values were normalized against the housekeeping gene (*Pipia*). The data are represented as the mean ± SD, ∗∗*p* < 0.01 and ∗∗∗*p* < 0.001. The scale bar represents 100 μm. ns, no significance; smad2-βKO, deletion of smad2 protein in *ins1*^*cre*^*;smad2*^*fx/fx*^.

Mice carrying conditional *smad2*^*fx/fx*^ alleles (18) were crossed with *ins1*^*cre*^ transgenic mice, expressing *cre*-recombinase in β-cells under control of the *ins1* promoter (Fig. 1*A*) (19). Deletion of smad2 protein in *ins1*^*cre*^*;smad2*^*fx/fx*^ (smad2-βKO) mice was confirmed by Western blot analysis (Fig. 1*B*) and immunohistochemistry (IHC) (Fig. 1*C*), showing a significant decrease in the amount of smad2 protein in the islets. Next, we examined the expression of smad2 mRNA in the isolated smad2-βKO islets by real-time PCR (RT-PCR) (Fig. 1*D*). We were able to detect some smad2 mRNA expression in the smad2-βKO islets, likely because of the presence of endothelial cells, pericytes, and non–β-endocrine cells (alpha, gamma, and delta cells), which are all *ins1*^*cre*^ negative. Moreover, there was normal smad2 mRNA expression in the liver and skeletal muscles of smad2-βKO mice (Fig. 1*D*), demonstrating that the deletion of smad2 is specific to β-cells.

### Improved glucose tolerance and glucose stimulated insulin secretion in smad2-βKO mice and isolated islets

**Figure 2:**
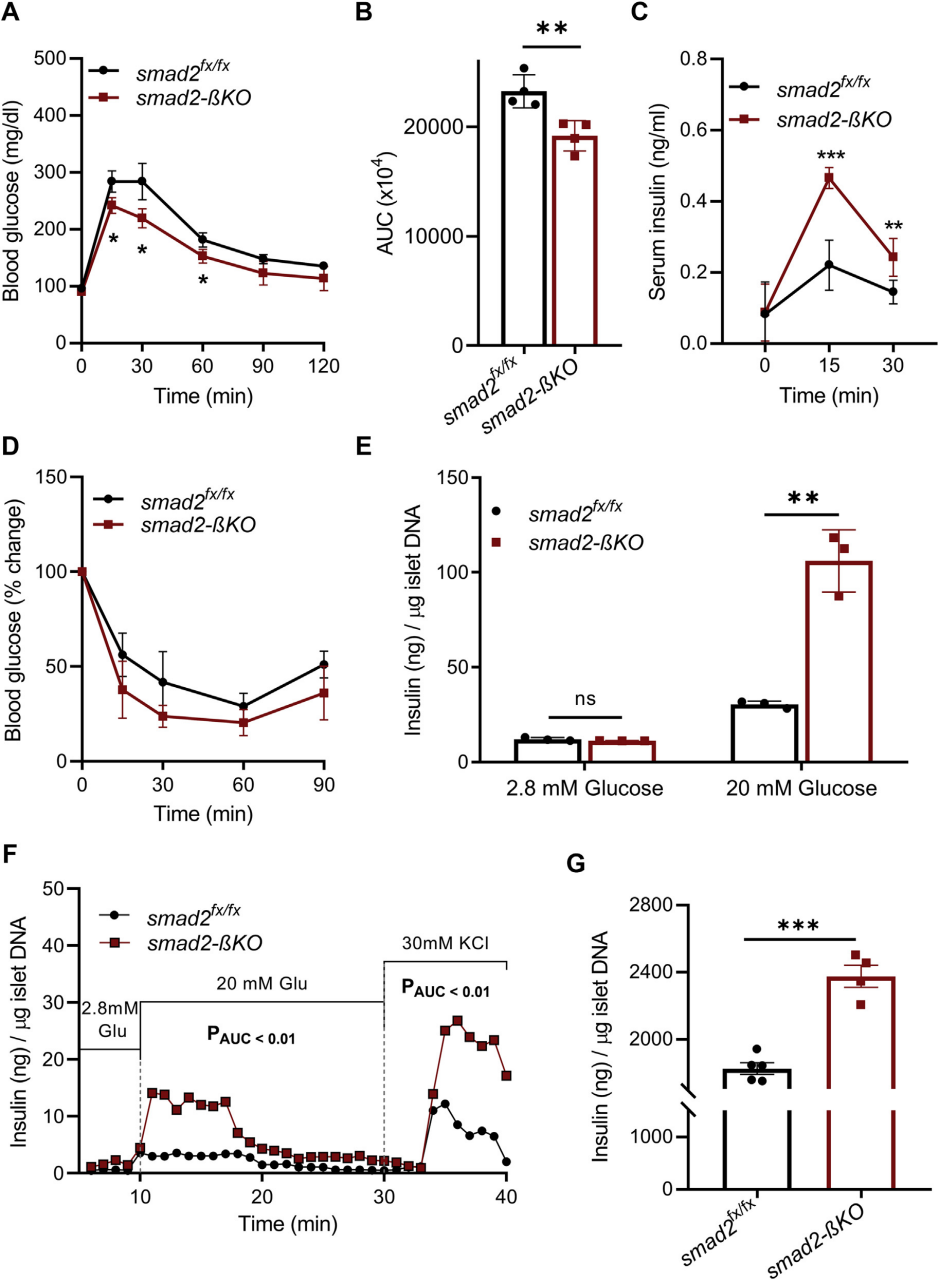
**Improved glucose tolerance and GSIS in smad2-βKO mice and isolated islets.***A*, IPGTT was performed for 14-week-old female mice, showing improved glucose tolerance in the smad2-βKO mice (*red squares*) compared with their littermate controls (*black circles*); n = 4 per group. *B*, analysis of the area under the curve (AUC) for the IPGTT. *C*, *in vivo* GSIS was performed for the 14-week-old littermate controls (*black circles*) and smad2-βKO mice (*red squares*); n = 4 per group. Smad2-βKO exhibited increased serum insulin levels at 15 and 30 min compared with their littermate controls. *D*, IPITT in 14-week-old mice did not show a significant difference between smad2-βKO mice (*red squares*) and their littermate controls (*black circles*); n = 4 per group. *E*, *ex vivo* static GSIS on isolated islets from 14-week-old mice. Islets isolated from the smad2-βKO mice (*red squares*) showed higher insulin release in response to the high glucose concentration (20 mM) than their controls (*black circles*); n = 3 mice per group, 30 islets/mouse. *F*, *ex vivo* islet perifusion assay with islets harvested from 14-week-old controls (*black circles*, n = 3 mice, 50 islets/mouse) and smad2-βKO mice (*red squares*, n = 4 mice, 50 islets/mouse). Islets from the smad2-βKO mice showed higher insulin release in response to a high glucose concentration (20 mM) and KCl perifusion. The differences between the two groups in each of the three conditions (low glucose, high glucose, and KCl) were analyzed by the AUC followed by unpaired Student's *t* test. Only significant *p*-values are depicted. *G*, insulin content was compared between islets isolated from 14-week-old controls (*black circles*, n = 5) and smad2-βKO mice (*red squares*, n = 4). The insulin content was higher in smad2-βKO islets. The data are represented as the mean ± SD, ∗*p* < 0.05, ∗∗*p* < 0.01, and ∗∗∗*p* < 0.001. GSIS, glucose-stimulated insulin secretion; IPITT, intraperitoneal insulin tolerance testing; IPGTT, intraperitoneal glucose tolerance test; ns, no significance; smad2-βKO, deletion of smad2 protein in *ins1*^*cre*^*;smad2*^*fx/fx*^.

The Smad2-βKO mice were viable and fertile with body weights comparable with their littermates for up to 1 year of observation. The smad2-βKO mice showed improved glucose tolerance (Fig. 2, *A* and *B*) and enhanced insulin secretion compared with their littermate controls (Fig. 2*C*). However, there was no significant difference in the insulin sensitivity (intraperitoneal insulin tolerance testing [IPITT]) between the smad2-βKO mice and controls (Fig. 2*D*), which suggests that the increased insulin secretion in the smad2-βKO mice is the primary cause for the improved glucose tolerance.

We then studied glucose-stimulated insulin secretion (GSIS) in the smad2-βKO isolated islets using a static assay and an islet perifusion assay to assess dynamic insulin release. In the static GSIS, the smad2-βKO islets showed enhanced insulin release in response to a high glucose concentration (Fig. 2*E*). Similarly, in the dynamic perifusion assay, there was a significant increase in insulin secretion in response to a high glucose concentration, with a robust burst of insulin release in response to KCl (Fig. 2*F*). To determine whether the increased insulin secretion correlates with greater insulin synthesis, we measured the insulin content of islets isolated from the smad2-βKO mice. The insulin content was significantly increased in the smad2-βKO islets compared with that of controls (Fig. 2*G*). These data suggest that smad2 may have an inhibitory effect on insulin synthesis and insulin secretion in response to glucose, both *in vivo* and *ex vivo.*

### Loss of smad2 in β-cells enhances expression of genes involved in β-cell function and increases β-cell mass and proliferation

**Figure 3:**
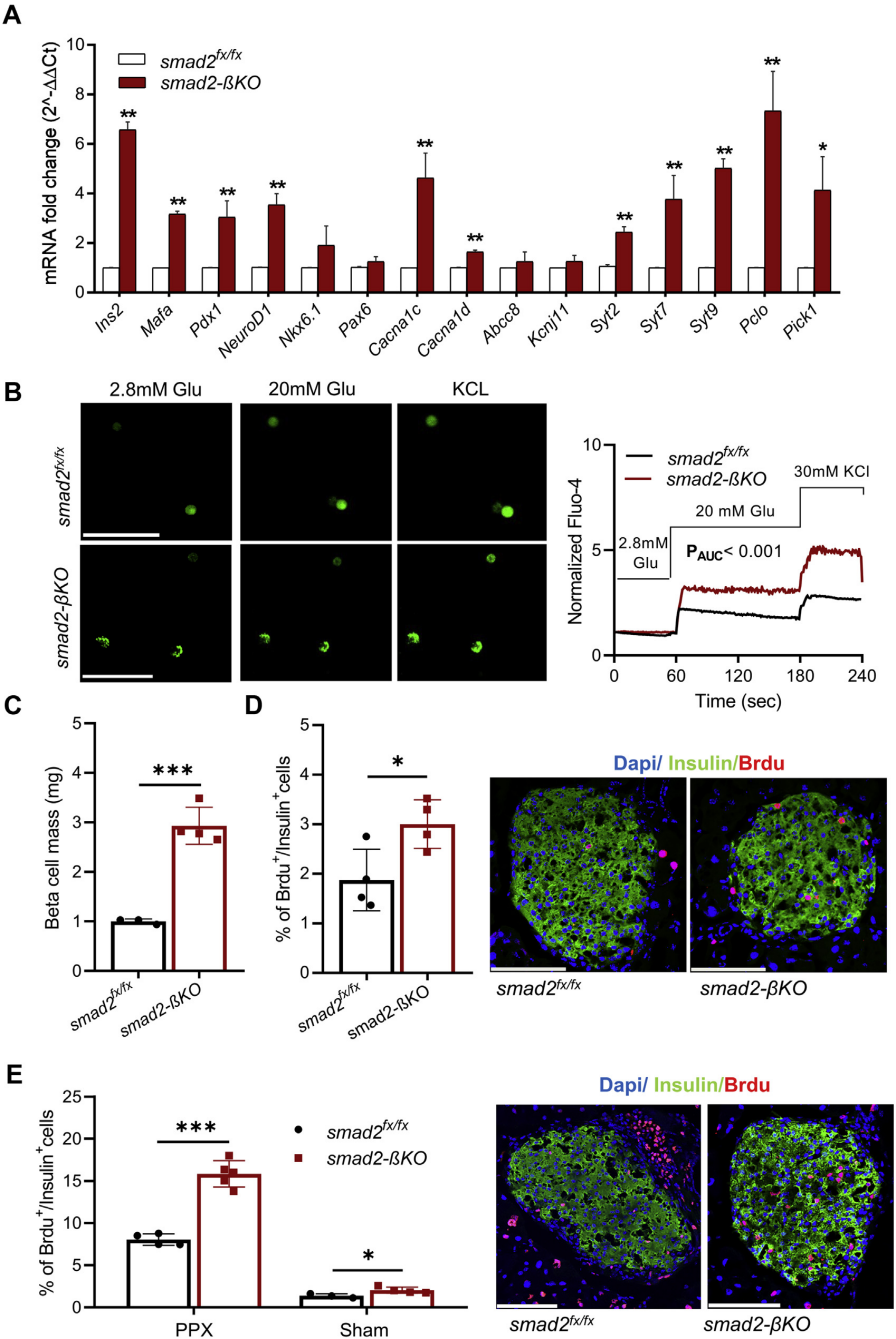
**Loss of smad2 in β-cells enhances expression of genes involved in β-cell function and increases β-cell mass and proliferation**. *A*, expression levels of insulin 2 gene, certain β-cell differentiation markers, ATP-sensitive potassium channel subunits (*Abcc8* and *Kcnj11*), voltage-gated calcium channel subunits alpha1C and alpha1D (*Cacna1c* and *Cacna1d*), Ca^2+^ channel, and insulin secretion–specific mRNAs; synaptotagmin-2, synaptotagmin-7, and synaptotagmin-9 (*Syt2*, *Syt7*, and *Syt9*), Piccolo (*Pclo*), and PICK1(*Pick1*) were all quantified by RT-PCR in cells from isolated islets of the 14-week-old *smad2*^*fx/fx*^ (*white bars*, n = 3) and smad2-βKO mice (*red bars*, n = 3). Values were normalized against the housekeeping gene (*Pipia*), with the latter being consistent across all conditions. *B*, confocal images of dispersed islet cells labeled with Fluo-4 (*left panel*). Normalized mean Fluo-4 intensities were analyzed (*right panel*), showing that smad2-βKO mice (*red curve*) had increased intensity of Fluo-4 after high glucose concentration (20 mM) and KCl stimulation compared with their littermate controls (*black curve*); the differences between the two groups were analyzed by the AUC followed by unpaired Student's *t* test, with *p* < 0.001. *C*, calculation of β-cell mass in the 14-week-old smad2-βKO mice (*red squares*, n = 4) and their littermate controls (*black circles*, n = 3), showing a significant increase in β-cell mass in smad2-βKO mice compared with their littermate controls. *D*, representative images (*right panel*) and quantification (*left panel*) of coimmunostaining for insulin and BrdU in the 14-week-old smad2-βKO mice (*red squares*) and their littermate controls (*black circles*); n = 4 per group, islets from the smad2-βKO mice had higher BrdU^+^/Ins^+^ cells than those from the control mice. *E*, partial pancreatectomy (PPX) or sham surgery was performed on the 14-week-old smad2-βKO mice (*red squares*, n = 5 in the PPX group and n = 4 in the sham group) and their littermate controls (*black circles*, n = 4 in the PPX group and n = 3 in the sham group). One week after surgery, β-cell proliferation was determined by the BrdU incorporation assay. Representative images (*right panel*) and quantification (*left panel*) showed that smad2-βKO islets had higher BrdU^+^/Ins^+^ cells than their littermate controls. Data are represented as the mean ± SD, ∗*p* < 0.05, ∗∗*p* < 0.01, and ∗∗∗*p* < 0.001. The scale bar represents 100 μm. BrdU, bromodeoxyuridine; RT-PCR, real-time PCR; smad2-βKO, deletion of smad2 protein in *ins1*^*cre*^*;smad2*^*fx/fx*^.

We investigated the role of smad2 in the expression of the insulin gene and transcription factors that are crucial for β-cell function. By RT-PCR, islets isolated from the smad2-βKO mice had significantly higher expression of insulin2 mRNA than those from their littermate controls (Fig. 3*A*). In addition, factors that directly regulate insulin gene expression, including MafA, Pdx1, and NeuroD1 (20) are upregulated in the smad2-βKO islets compared with controls (Fig. 3*A*). In keeping with the RT-PCR data, immunostaining of pancreas sections showed increased percentages of Mafa^+^/Insulin^+^, Pdx1^+^/Insulin^+^, and NeuroD1^+^/Insulin^+^ cells in smad2-βKO (Fig. S1). However, Nkx-6.1 (by immunostaining and RT-PCR), a transcription factor required for maintaining β-cells in their differentiated state (21), and Pax-6 (by RT-PCR), a transcription factor required for β-cell differentiation and optimal β-cell function (22), were similar in the smad2-βKO islets and the controls (Figs. 3*A* and S1).

Next, we investigated the effect of smad2 loss on the expression of potassium and calcium channels known to be involved in GSIS (23, 24). The mRNA expression of ATP-sensitive potassium channel subunits (*Kcnj11* and *Abcc8*) was similar in the smad2-βKO islets and controls. However, the mRNA expression of (1) *Cacna1c* and *Cacna1d* (voltage-gated calcium channel subunits alpha1C and alpha1D), (2) *synaptotagmin*-*2*, *synaptotagmin*-*7*, and *synaptotagmin*-*9* and *piccolo* (calcium sensor genes that facilitate calcium-induced exocytosis) (20, 25, 26), and (3) *PICK1* (a PDZ domain–containing peripheral membrane protein that regulates the trafficking of insulin granules) (27) was higher in smad2-βKO islets than controls (Fig. 3*A*).

Furthermore, we imaged the calcium flux in dispersed islet cells using the calcium indicator Fluo-4. The smad2-βKO islet cells showed significantly increased Fluo-4 fluorescence in response to high glucose and KCl compared with control islet cells, indicating increased calcium influx (Fig. 3*B*).

Given the improved glucose tolerance observed in the smad2-βKO mice, we examined the effect of smad2 loss on β-cell mass. Remarkably, the smad2-βKO mice showed a higher β-cell mass than controls (Fig. 3*C*). To test whether the increased β-cell mass is due to enhanced proliferation, mice were treated with bromodeoxyuridine (BrdU) in the drinking water for 1 week to label proliferating β-cells. Quantification of BrdU^+^/insulin^+^ percentages showed a 1.6-fold increase in baseline β-cell proliferation in smad2-βKO compared with controls (Fig. 3*D*).

To further confirm that smad2 loss increases β-cell proliferation, we used 60% PPX , a model for workload-induced β-cell proliferation. After PPX, which increased β-cell proliferation 4-fold in the control mice, there was a further 2.6-fold increase in the number of BrdU^+^ cells in the smad2-βKO mice compared with the controls (Fig. 3*E*).

These data suggest that the overall suppression of TGF-β/smad2 signaling increases insulin synthesis, upregulates transcription factors involved in insulin gene expression, and induces β-cell proliferation.

### Loss of smad2 in β-cells improves HFD-induced hyperglycemia and improves GSIS *in vivo* and in isolated islets

**Figure 4:**
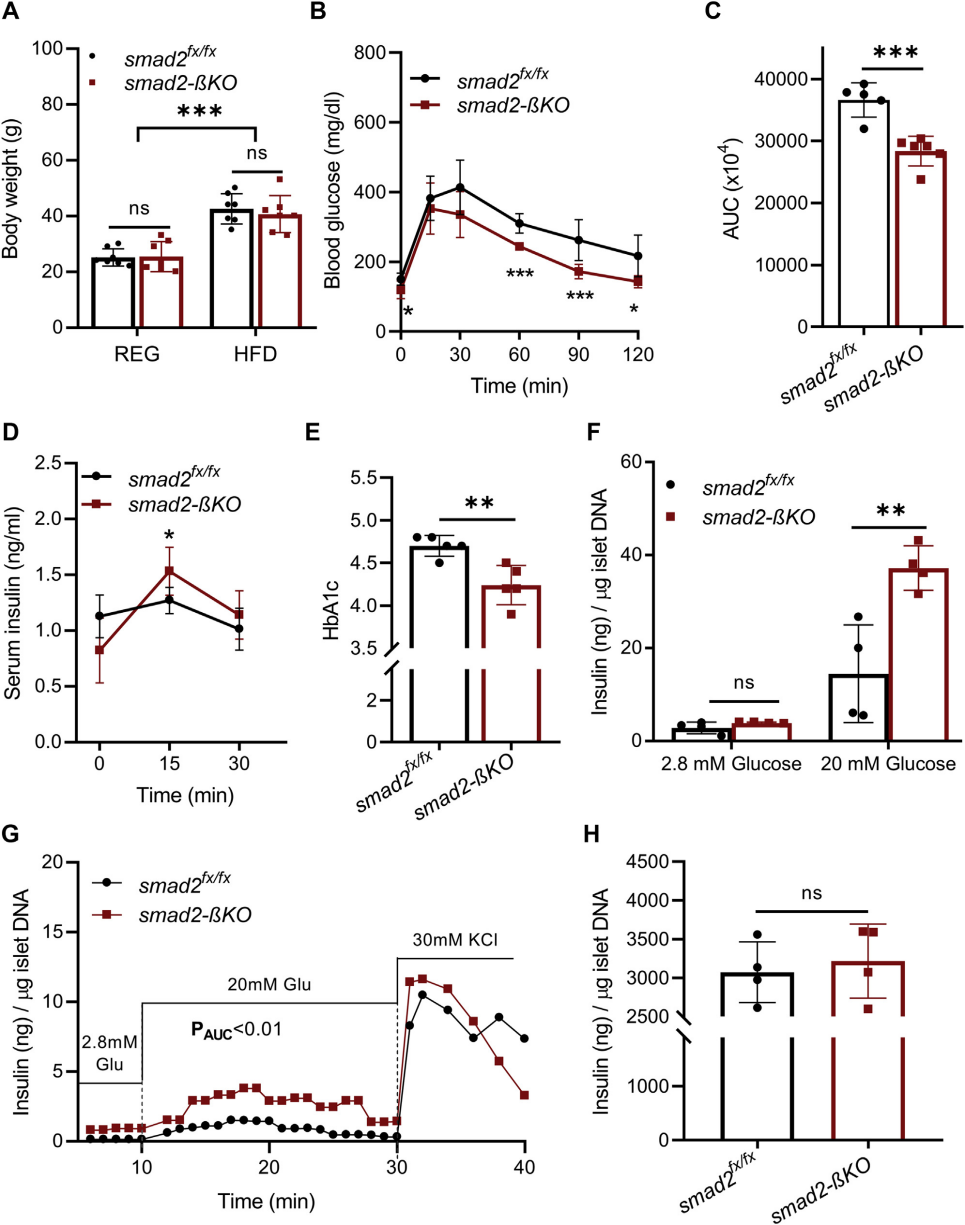
**Loss of smad2 in β-cells improves HFD-induced hyperglycemia and improves GSIS *in vivo* and in isolated islets.***A*, body weight of 18-week-old smad2-βKO mice (*red squares*) and their littermate controls (*black circles*) on regular chow (REG) or 60% high-fat diet (HFD); n = 7 per group. *B*, after 12 weeks of HFD feeding, IPGTT was performed for 18-week-old female mice, showing improved glucose tolerance in smad2-βKO mice (*red squares*, n = 6) compared with their littermate controls (*black circles*, n = 5). *C*, analysis of the AUC for the IPGTT. *D*, *in vivo* GSIS was carried out after 12 weeks of HFD, for smad2-βKO mice (*red squares*) and littermate controls (*black circles*); n = 6 per group. Compared with the controls, smad2-βKO mice exhibited significant increase in serum insulin levels at 15 min. In addition, the fasting insulin levels were lower in smad2-βKO mice (*p* = 0.057). *E*, after 12 weeks of HFD feeding, HbA1c was significantly lower in smad2-βKO mice (*red squares*) than their littermate controls (*black circles*); n = 5 per group. *F*, *ex vivo* GSIS on isolated islets from the 18-week-old HFD-fed mice. The islets isolated from smad2-βKO mice (*red squares*) showed higher insulin release in response to high glucose concentration (20 mM) than those from their controls (*black circles*); n = 4 mice per group, 30 islets/mouse. *G*, *ex vivo* islet perifusion assay with islets harvested from the 18-week-old smad2-βKO mice (*red squares*) and their littermate controls (*black circles*), after 12 weeks of HFD feeding. The islets from smad2-βKO showed higher insulin release in response to high glucose concentration (20 mM). The differences between the two groups in each of the three conditions (low glucose, high glucose, and KCl) were analyzed by the AUC followed by unpaired Student's *t* test. N = 3 mice per group, 50 islets/mouse. Only significant *p*-values are depicted. *H*, after 12 weeks of HFD, the insulin content was compared between islets isolated from the 18-week-old smad2-βKO *mice* (*red squares*) and their control littermates (*black circles*); n = 4 per group. There was no significant difference between the two groups. All data are represented as the mean ± SD; ∗*p* < 0.05, ∗∗*p* < 0.01, and ∗∗∗*p* < 0.001. AUC, area under the curve; GSIS, glucose-stimulated insulin secretion; IPGTT, intraperitoneal glucose tolerance test; ns, no significance; smad2-βKO, deletion of smad2 protein in *ins1*^*cre*^*;smad2*^*fx/fx*^.

HFD-induced obesity in C57BL/6J mice mirrors the human metabolic derangements of obesity (28, 29, 30). We put 6-week-old smad2-βKO mice and their littermate controls on 60% HFD or regular chow for 12 weeks. The HFD mice had significantly increased weight gain compared with the controls, but there was no difference in the body weight between smad2-βKO mice and their littermates within either feeding regimens (Fig. 4*A*). After 12 weeks of HFD, the HFD–smad2-βKO mice showed improved glucose tolerance (Fig. 4, *B* and *C*) and increased insulin secretion (Fig. 4*D*) compared with the HFD controls. It was also observed that the HFD–smad2-βKO mice had lower fasting insulin levels than the HFD controls (Fig. 4*D*), possibly indicating an improvement in the abnormally increased endogenous hepatic glucose production reported in this model (31). Furthermore, to investigate the effect of β-cell-specific smad2 loss on long-term glucose homeostasis, we measured glycated hemoglobin (HbA1c). The HFD–smad2-βKO mice exhibited a slight but significant decrease in HbA1c compared with the HFD controls (Fig. 4*E*).

Next, we analyzed islets isolated from the HFD–smad2-βKO mice and their littermate HFD controls after 12 weeks of HFD. In line with the *in vivo* data, islets isolated from the HFD–smad2-βKO mice showed increased insulin release in response to high glucose in both static GSIS (Fig. 4*F*) and dynamic perifusion studies (Fig. 4*G*), but no difference was observed between the two groups in insulin release after KCl stimulation during the dynamic perifusion study (Fig. 4*G*). Similarly, there was no significant difference observed in the insulin content between the islets isolated from the HFD–smad2-βKO mice and the HFD controls (Fig. 4*H*).

### Loss of smad2 improves insulin sensitivity markers in the liver and peripheral tissues in HFD mice

**Figure 5:**
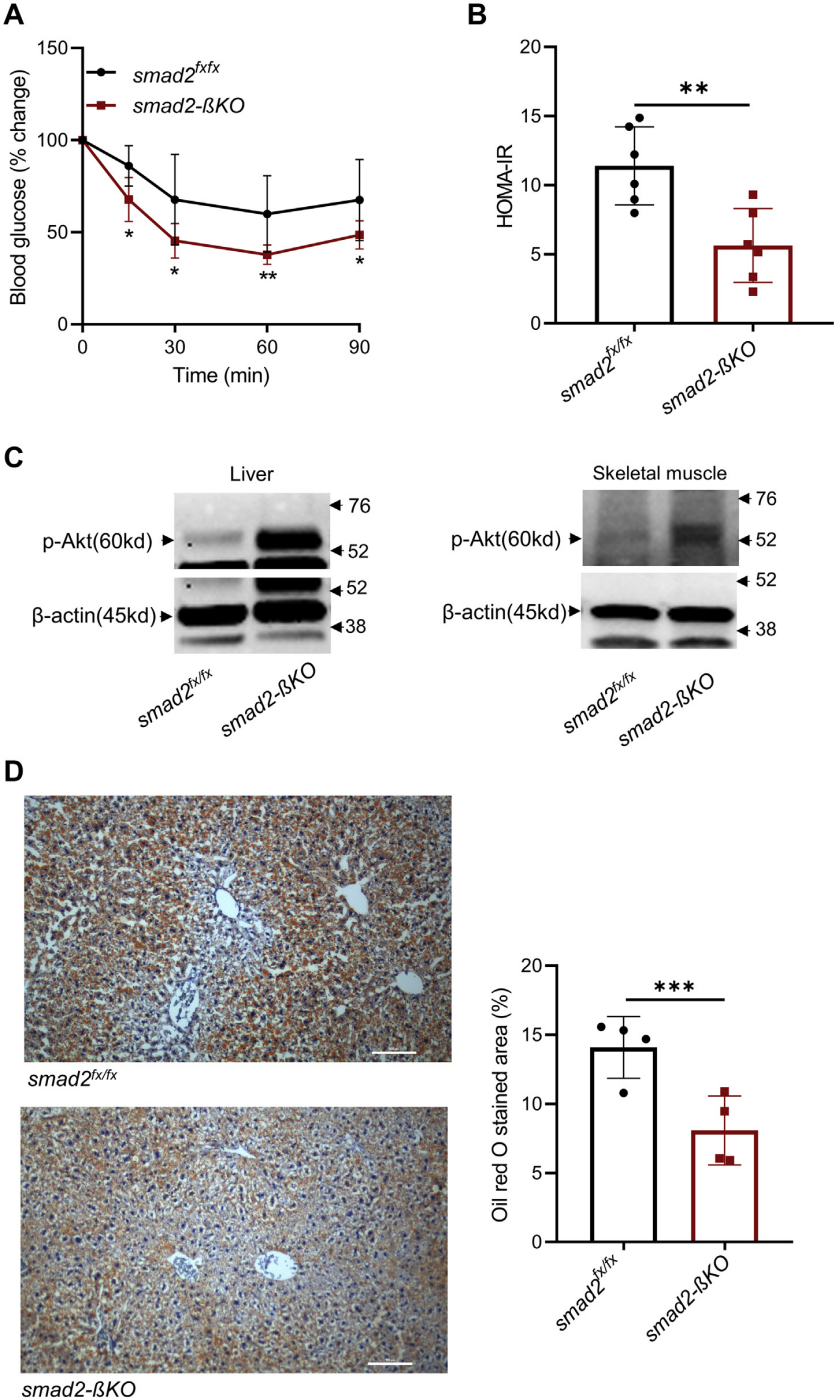
**Loss of smad2 improves insulin sensitivity in HFD mice.***A*, IPITT after 12 weeks of HFD showed a significant improvement in insulin sensitivity in the HFD–smad2-βKO mice (*red squares*, n = 9) compared with their littermate HFD controls (*black circles*, n = 6). Data are presented as the percent change in blood glucose from the fasting level. *B*, calculated HOMA-IR in the HFD–smad2-βKO mice (*red squares*) and their littermate HFD controls (*black circles*); n = 6 per group. *C*, immunoblot analysis of p-Akt (Ser473) in the liver and skeletal muscle of smad2-βKO mice and littermate controls, after 12 weeks of HFD. Cropped gels are displayed. Smad2-βKO mice had higher expression of p-Akt in the liver and skeletal muscle than their littermate controls. β-Actin was used as a loading and transfer control. *D*, representative images (*left panel*) and quantification for Oil-Red-O staining of liver specimens (*right panel*) showed significantly lower hepatic fat in smad2-βKO than their littermate controls after 12 weeks of HFD (n  = 4 per group). All data are shown as the mean ± SD. ∗*p* < 0.05, ∗∗*p* < 0.01, and ∗∗∗*p* < 0.001. The scale bar represents 100 μm. HFD, high-fat diet; HOMA-IR, homeostatic model assessment–estimated insulin resistance; p-Akt, phosphorylated Akt; smad2-βKO, deletion of smad2 protein in *ins1*^*cre*^*;smad2*^*fx/fx*^.

The HFD–smad2-βKO mice had improved insulin sensitivity compared with the HFD controls, as measured by IPITT (Fig. 5*A*). In support of IPITT results, calculation of the homeostatic model assessment–estimated insulin resistance (HOMA-IR) index, a surrogate marker for insulin resistance (32), showed that the HFD–smad2-βKO mice had a significantly lower HOMA-IR index than the HFD controls (Fig. 5*B*). To further investigate the improved insulin sensitivity in HFD–smad2-βKO mice, we examined the protein expression levels of phosphorylated Akt, a key mediator of insulin-receptor signaling (33). After 12 weeks of HFD, there was an increased phosphorylated Akt protein expression in the liver and skeletal muscle of HFD–smad2-βKO mice when compared with the HFD controls (Fig. 5*C*).

We also studied the effect of smad2-βKO on hepatic steatosis, which is related to increased hepatic insulin resistance in the HFD mice (34). Quantification of the hepatocyte lipid-droplet accumulation (Fig. 5*D*) showed a significantly lower percent area of liver lipid droplets in HFD–smad2-βKO mice than the HFD controls, indicating that the loss of smad2 improves hepatic steatosis in the HFD mice.

### Loss of smad2 in β-cells decreases endoplasmic reticulum stress and increases β-cell proliferation and β-cell mass in HFD mice

**Figure 6:**
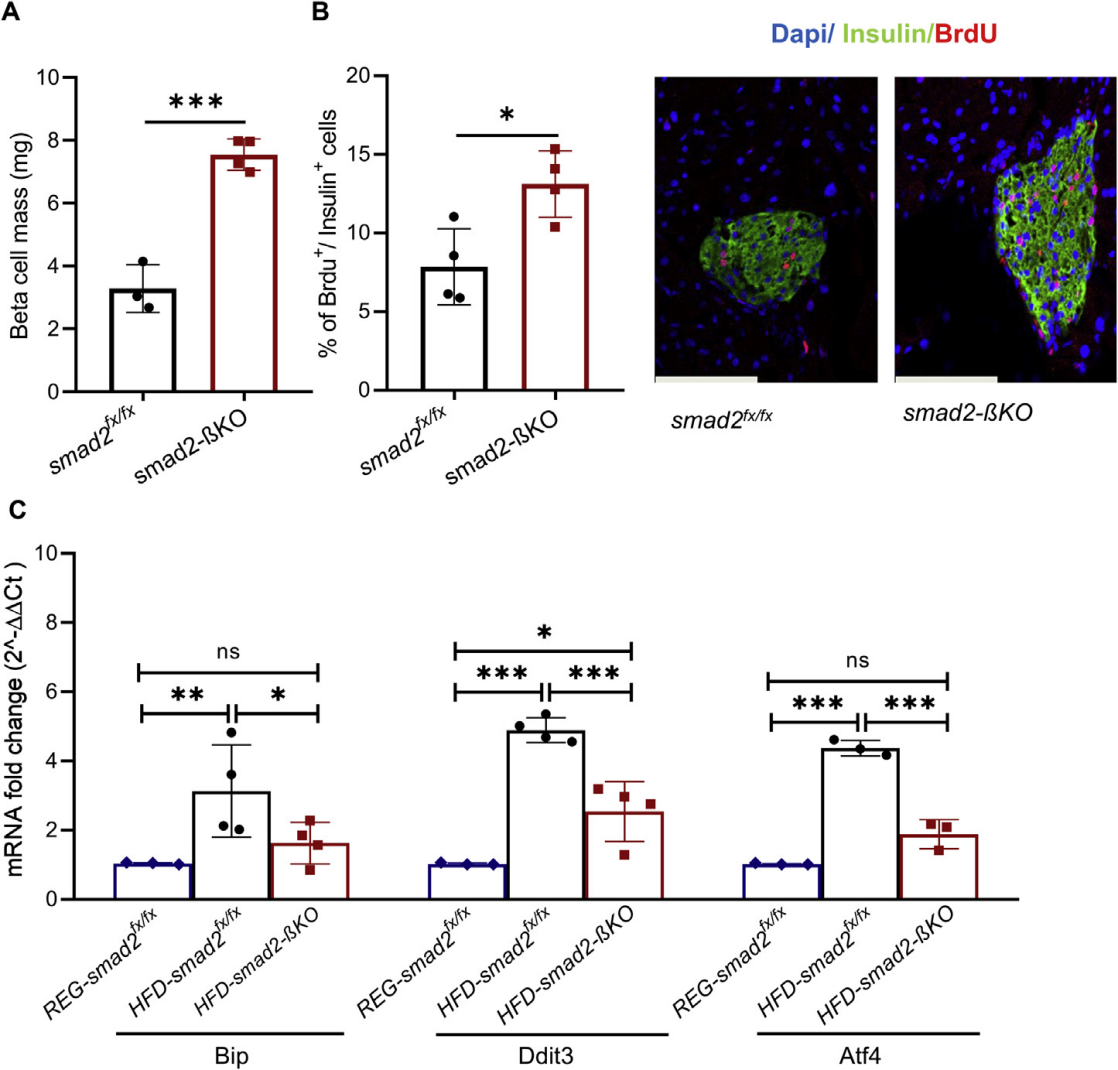
**Loss of smad2 in β-cells decreases ER stress and increases β-cell proliferation andβ-cellmass in HFD-fed mice.***A*, calculation of β-cell mass in smad2-βKO mice (*red squares*, n = 4) and littermate controls (*black circles*, n = 3), after 12 weeks of HFD, showing a significant increase in the β-cell mass in smad2-βKO mice. *B*, representative images (*right panel*) and quantification of coimmunostaining for insulin and BrdU (*left panel*) in the smad2-βKO mice (*red squares*) and littermate controls (*black circles*), after 12 weeks of HFD; n = 4 per group. The Smad2-βKO islets had higher BrdU^+^/Ins^+^ cells than the control mice. *C*, the expression levels of certain ER-stress markers (*Bip*, *Ddit3*, and *Atf4*) were quantified by RT-PCR in islets isolated from 18-week-old control mice on regular diet (*blue diamonds*, n = 3), HFD control (*black circles*, n = 4), and HFD–smad2-βKO mice (*red squares*, n = 4). The islets isolated from HFD–smad2-βKO mice showed a significantly lower expression of ER-stress markers than those from the HFD controls. The values were normalized against the housekeeping gene (*Pipia*), with the latter being consistent across all conditions. The data are represented as the mean ± SD, ∗*p* < 0.05, ∗∗*p* < 0.01, and ∗∗∗*p* < 0.001. The scale bar represents 100 μm. BrdU, bromodeoxyuridine; ER, endoplasmic reticulum; HFD, high-fat diet; ns, no significance; smad2-βKO, deletion of smad2 protein in *ins1*^*cre*^*;smad2*^*fx/fx*^; RT-PCR, real-time PCR.

To investigate the possible mechanisms behind the improved adaptation of the smad2-βKO mice to diet-induced metabolic stress, we examined the changes in β-cell mass after 12 weeks of HFD. We found a significant increase in the calculated β-cell mass in the HFD–smad2-βKO mice compared with the HFD controls (Fig. 6*A*). To examine whether there was a corresponding increase in β-cell proliferation in HFD–smad2-βKO mice, we analyzed BrdU labeling of β-cells in the HFD mice; we found a 1.7-fold increase in BrdU+ β-cells in HFD–smad2-βKO mice compared with the HFD controls (Fig. 6*B*). Because long-term HFD in mice is reported to cause β-cell failure and increased endoplasmic reticulum (ER) stress (30, 35, 36), we, therefore, investigated the effect of smad2 deletion on ER stress in β-cells. The expression of various ER-stress markers in isolated islets was assessed by RT-PCR (*BiP*, *Ddit*, and *Atf4*). Remarkably, islets isolated from the HFD–smad2-βKO mice showed a significant decrease in the expression of ER-stress markers compared with the HFD controls; moreover, the expression of some ER-stress markers in the HFD–smad2-βKO islets was nearly similar to that in islets from regular chow controls (Fig. 6*C*). Furthermore, we studied the effect of smad2 deletion on the expression of ER-stress markers at baseline conditions (regular chow); we found decreased expression of ER stress–related genes (*BiP*, *Ddit3*, and *Atf4*) (Fig. S2), without a significant change in the protein levels of p-PERK or p-elF2α when compared with the controls on regular chow (Fig. S2).

## Discussion

In the present study, we have shown that loss of smad2 in β-cells improves glucose tolerance, enhances insulin secretion, and increases β-cell mass at baseline and with HFD.

Insulin release in response to glucose is predominantly a function of the total number of β-cells in the pancreatic islets (β-cell mass) and the capability of each of these β-cells to secrete insulin (37). In addition, insulin secretion in an individual β-cell is regulated mainly at two levels, the level of exocytosis that involves signaling pathways mediating insulin granule trafficking and insulin release (26) and at the level of gene transcription and translation, which regulate insulin biosynthesis (20). In the present study, we showed that loss of smad2 led to upregulation of expression of insulin (with increased insulin content, Fig. 2*G*), insulin transcription factors, and factors involved in insulin secretion and exocytosis (Fig. 3*A*). These findings all together can well explain the improved β-cell function in this model (Fig. 2). Notably, the improvement in insulin secretion in smad2-βKO mice on normal diet (Fig. 2) was more pronounced than its improvement in smad2-βKO mice on HFD (Fig. 4) when compared with their respective controls. This lesser improvement in HFD β-cells may reflect a prolonged exposure to glucolipotoxicity, which negatively impacts β-cell function (38, 39, 40, 41).

Our finding that blocking smad2 signaling is associated with improved β-cell function is consistent with previous studies using a global smad3 KO mouse model or treatment with TGF-β inhibitory molecules (15, 17). In addition, smad proteins (smad2, smad3, and smad4) were reported to inhibit transactivation of insulin by binding and inactivating MafA protein (16). Together, our data and those of others suggest that smad2 and smad3 could be therapeutic targets to treat diabetes. Further studies are required to compare the effect of smad2 and smad3 deletion on β-cell proliferation and β-cell function.

Another study appeared contradictory to our findings, where Rip-cre/smad2^fx/fx^ mice (RIP for “rat insulin promoter”) showed impaired glucose tolerance, impaired insulin secretion, and islet hyperplasia (42). In the mouse model used in that study, human growth hormone is synthesized and secreted from the Rip-cre islets, causing cre-independent alterations in β-cell function (43). In addition, several reports have shown that Rip-cre mice alone exhibit glucose intolerance (43, 44, 45), likely as a result of the use of the powerful RIP promoter, which leads to extremely high production of cre, and thus potentially causing or exacerbating ER-stress. In addition, the *ins1*^*cre*^ mouse model used in our study has been shown to induce effective and selective recombination of floxed genes in β-cells, without recombination in the central nervous system (19), as opposed to Rip-cre mice, which are known to express cre in the hypothalamus (46, 47), which may affect the weight and the feeding behavior of these transgenic mice (42). These issues all raised concerns about the cell-type specificity and the interpretation of the phenotypes found in Rip-cre mice (48).

Another significant finding in the present study is that the loss of smad2 specifically in β-cells led to increased β-cell mass and proliferation (Fig. 3, *C* and *D*). This finding is consistent with our previous reports which shows that the inhibition of TGF-β signaling either by smad2/smad3 KO or enhanced expression of the smad inhibitor, smad7, resulted in an increased number of proliferating β-cells (11, 12, 13). We also demonstrated that PPX in smad2-βKO mice led to robust β-cell proliferation, which suggests that TGF-β/smad2 signaling has a significant negative effect on β-cell proliferation when there is an increase in the workload demand.

In view of our initial findings, we proceeded to examine the effect of smad2 deletion in an HFD-fed C57BL/6 mouse model that mirrors the metabolic derangements that occur in humans with obesity (29, 30). In line with the data obtained in mice fed regular chow, loss of smad2 in the HFD mice improved glucose tolerance, insulin secretion, and increased β-cell mass. Moreover, smad2-βKO mice had improved insulin sensitivity and ameliorated hepatic steatosis, despite continued HFD. The increase in insulin sensitivity in the smad2-βKO HFD mice likely contributed to the improvement in hepatic steatosis, as it is known that fatty liver can be directly related to insulin resistance (49, 50).

In addition, the improved β-cell function observed in the smad2-βKO obese mice was associated with decreased ER-stress markers in the isolated islets (Fig. 6*C*), suggesting that blocking TGF-β/smad signaling is protective against diet-induced ER stress in β-cells. In support of this finding, previous studies showed that stimulation of TGF-β is associated with increased ER stress in the lung and liver cells (51, 52); however, more studies are needed to understand the roles of the TGF-β signaling pathway in diet-induced ER stress in pancreatic islets.

In conclusion, our study provides evidence that smad2 plays a vital role in regulating β-cell function and proliferation. The physiological role of the suppression of insulin biosynthesis by TGF-β/smad is still unclear. One could speculate that repressing superfluous insulin biosynthesis and restraining proliferation helps maintain circulating insulin levels in the normal range (53). However, under metabolic stress, TGF-β/smad signaling could be harmfully suppressing an adaptive capacity of β-cells, thus adding to the deleterious effect of ER stress, which can eventually lead to β-cell failure. Our study thus suggests that inhibiting TGF-β/smad signaling may be a potential therapeutic target for T2DM.

## Experimental procedures

### Mouse manipulations

Floxed smad2 knock-in (*smad2*^*fx/fx*^) and *insulin1*^*cre*^ mice in a pure C57BL/6J background were obtained from the Jackson Laboratory . *Ins1*^*cre*^ mice were crossed with *smad2*^*fx/fx*^ to generate β-cell-specific smad2 KO mice (smad2-βKO). Equal numbers of 14-week-old males and females were used in each group. For the HFD experiments, 6-week-old mice were placed on regular chow or HFD (60% kcal from fat, D12492; Research Diets) for 12 weeks. All animals were housed under specific pathogen-free conditions in the animal facility at the Children's Hospital of Pittsburgh. The Animal Research and Care Committee at the Children's Hospital of Pittsburgh and the University of Pittsburgh Institutional Animal Care and Use Committee reviewed and approved all the mouse experiments described in this study.

### *In vivo* glucose homeostasis studies

#### Intraperitoneal glucose tolerance test

Sixteen-hour–fasted mice were intraperitoneally injected with 2 g/kg glucose (Sigma-Aldrich). Blood glucose levels were measured at 0, 15, 30, 60, 90, and 120 min after the glucose injection using a glucometer (Contour next EZ).

#### GSIS

Serum insulin concentrations were measured during intraperitoneal glucose tolerance test. Approximately 50 μl blood was collected from the tail vein at 0, 15, and 30 min *via* Microvette CB 300Z, clotting activator/serum tubes (Sarstedt). Serum insulin was measured using a mouse insulin ELISA kit (80-INSMSU-E01, ALPCO).

#### IPITT

Six-hour–fasted mice were intraperitoneally injected with (0.75 U/kg) insulin (Humulin, Lilly), and blood glucose was measured at 0, 15, 30, 60, and 90 min after insulin injection.

#### HOMA-IR index

The HOMA-IR index was calculated using the following formula: HOMA-IR = [fasting glucose (mmol/l) × fasting insulin (mU/l)] ÷ 22.5 (32).

### Measurement of HbA1c

Approximately 10 μl of blood was collected from the tail vein in conscious mice to measure HbA1c using the DCA Vantage Analyzer (Siemens). This system automatically measures both HbA1c and total hemoglobin, and the percent HbA1c is then calculated as follows: % HbA1c = ([HbA1c]/[Total Hemoglobin]) × 100. The ratio is reported as the percent HbA1c (54, 55).

### Pancreas digestion and islet isolation

Islets were isolated from the control and smad2-βKO mice as previously described (56). Briefly, the pancreatic duct was infused, and the pancreas was subsequently digested with type V collagenase (1.4 mg/ml). The islets were separated from the exocrine tissue with the Histopaque 1100 gradient solution (100 ml Histopaque 1077 and 120 ml Histopaque 1119) (Sigma-Aldrich) and then washed with Hanks' Balanced Salt Solution (Gibco) containing 20 mM Hepes buffer (Gibco) and 0.2% bovine serum albumin (Sigma-Aldrich). The islets were then handpicked to eliminate any contamination from exocrine tissue.

### Islet perifusion assay

The isolated islets were left to recover overnight at 37 °C (5% CO_2_) in the RPMI 1640 medium (Gibco) containing 10% fetal bovine serum. Then, groups of 50 islets per mouse were placed in a dynamic perifusion system (Amersham Biosciences AKTA FPLC System). The perifusion was performed using Krebs buffer with 2.8 mM glucose at a flow rate of 1 ml/min for 15 min to establish stable basal insulin secretion. Next, the islets were perifused with 2.8 mM glucose for 10 min, and fractions of 500 μl were collected every 30 s. Then, the glucose concentration was increased to 20 mM, and fractions of 500 μl were collected every 30 s for 20 min. Finally, the islets were perifused with 30 mM KCl, and fractions of (500 μl) were collected every 30 s for 10 min. After the perifusion, the islets were recollected from the column for genomic DNA measurement. The insulin in the effluent was measured by a mouse ELISA kit (ALPCO). The fractional insulin secretion rate was calculated as secreted insulin per minute normalized to the DNA content.

### *Ex vivo* static GSIS

The isolated islets were left to recover overnight at 37 °C (5% CO_2_) in the RPMI 1640 medium (Gibco) containing 10% fetal bovine serum. Then, groups of 30 islets per mouse were incubated in 2.8 mM glucose for 30 min at 37 °C to establish stable basal insulin secretion and then washed by Krebs buffer twice. The islets were transferred into a new well containing 2 ml of 2.8 mM glucose solution for 30 min at 37 °C, and 100-μl media was collected for time point 1 and then transferred into a new well containing 2 ml of 20 mM glucose solution for 30 min at 37 °C, and 100 μl media was collected for time point 2. The islets were then recovered for genomic DNA measurement. Insulin levels in the collected media were measured by the mouse ELISA kit (ALPCO) and normalized to the DNA content.

### Insulin content in the isolated islets

Ten equal-sized islets per mouse were incubated in 30 μl acid/ethanol (75% ethanol and 0.15 M HCl) at 4 °C overnight with gentle rotation to extract insulin and then centrifuged at 14,000 rpm for 10 min. The supernatant was diluted at 1:50 ratio, and the insulin content was measured by the mouse ELISA kit (ALPCO) and normalized to the total islet DNA content.

### Calcium imaging

The isolated islets were dispersed into single cells and plated on glass-bottom culture dishes (MatTek). The cells were loaded with 1 μM Fluo-4 (Thermo Fisher Scientific) at 37 °C for 30 min and then washed with Krebs buffer. The cells were incubated in 2.8 mM glucose for 1 min, exposed to 20 mM glucose for 2 min, and, finally, exposed to 20 mM KCl for 1 min. The intensity of Fluo-4 at 488 nm was monitored by live imaging of a 20× objective lens of a Zeiss LSM710 confocal microscope, and data were analyzed by ZEISS-ZEN software and normalized to the baseline intensity. Imaging was done for an average of four cells per field.

### Isolation of RNA and quantitative RT-PCR

Total RNA was extracted from isolated islets using the RNeasy Plus extraction kit (QIAGEN). RNA was reverse-transcribed into cDNA using iScript reverse transcription supermix (Bio-Rad). Quantitative gene expression was analyzed by using TaqMan Gene expression assays (Thermo Fisher Scientific) (Table S1). The ΔΔCt method was used to calculate relative fold changes of target genes with respect to the standard reference gene *Pipia*.

### Protein extraction

For protein extraction, the liver, skeletal muscle (quadriceps muscle), and adipose tissue (gonadal fat pad) samples were placed in approximately 600 μl of NP40 Cell Lysis Buffer (FNN0021) with 1 mM PMSF (36978) and protease inhibitor cocktail (1862209) (Thermo Fisher Scientific). After homogenization on ice, the tissue lysates were incubated on ice for 30 min. After centrifugation at 10,000*g* for 30 min at 4 °C, the supernatants were collected. Protein concentration was determined by bicinchoninic acid protein assay using QuantiPro BCA Assay Kit (Millipore-Sigma).

### Western blot

Lysates from isolated the islets, liver, and skeletal muscle were separated on SDS-PAGE gels and subsequently transferred to polyvinylidene fluoride membranes (Millipore). The membranes were blocked in 5% milk in tris-buffered saline with Tween 20 for 1 h and then incubated with rabbit monoclonal antibodies (Cell Signaling Technology) against total Smad-2/3 (8685S), Akt (4691S), Phospho-Akt (Ser473) (4060S), Phospho-PERK (Thr980) (3179S), Phospho-eIF2α (Ser51) (3398S), and β-actin (4970S) overnight at 4 °C. The membranes were washed and incubated with horseradish peroxidase–conjugated anti-rabbit (1705046, Bio-Rad) for 1 h. Bands were detected using Luminescent Image Analyzer LAS-3000. The density of each band was quantified using ImageJ software.

### IHC

Pancreas samples were fixed with 4% paraformaldehyde for 24 h at 4 °C, dehydrated using 30% sucrose overnight, embedded in optimal cutting temperature compound, snap-frozen by liquid nitrogen, and sectioned at 7 μm. For IHC, antigen retrieval was performed (heat and/or acid buffer). Slides were incubated with primary antibodies at 4 °C overnight and were then incubated with Fluorescent-conjugated (FITC, CY3) secondary antibodies (Jackson ImmunoResearch Labs) for 1 h at room temperature. Nuclear staining and mounting were performed using Fluoroshield with DAPI (Sigma-Aldrich).

### Oil-Red-O staining

Paraformaldehyde-fixed liver samples were dehydrated in 30% sucrose and snap-frozen with liquid nitrogen after embedding in optimal cutting temperature. Briefly, 7-μm sections were kept in PBS for 10 min and briefly washed with 60% isopropanol. Liver sections were placed in the Oil-red-O (O0625, Sigma-Aldrich) solution (0.5% in 60% isopropanol) for 30 min and then rinsed with 60% isopropanol to remove the nonspecific staining and counterstained with Mayer's hematoxylin (Sigma-Aldrich).

### Quantifications and data analysis

#### Quantification of β-cell proliferation and β-cell markers

In the insulin + cells, we manually quantified BrdU+, Pdx1+, MafA+, Nkx6.1+, and NeuroD1+ cells using ImageJ software from six (100 μm apart) sections per pancreas. The β-cell mass/area was quantified as described (12). Briefly, 10 sections at 100-μm intervals from the whole pancreas were immunostained for insulin and DAPI. Captured images of entire sections were analyzed using ImageJ software. The average β-cell mass was calculated by multiplying the pancreas weight with the insulin-positive:pancreas area ratio.

### Study approval

All mouse experiments were approved by the Animal Research and Care Committee at the Children's Hospital of Pittsburgh and the University of Pittsburgh Institutional Animal Care and Use Committee.

### Statistical analysis and calculations

All the data were analyzed using GraphPad Prism 8.3 (GraphPad Software). All values are depicted as the means ± SD. The differences between two groups in the intraperitoneal glucose tolerance , perfusion study, and Ca^2+^ tracing study were analyzed by using the area under the curve. All data were statistically analyzed by unpaired Student's *t* test, and statistically significant differences are shown for *p* < 0.05(∗), *p* < 0.01(∗∗), and *p* < 0.001(∗∗∗).

## Data availability

All data are contained within the article.

## Supporting information

This article contains supporting information.

## Conflict of interest

The authors declare that they have no conflicts of interest with the contents of this article.
