# Supplementary material for: β-cell Smad2 null mice have improved β-cell function and are protected from diet-induced hyperglycemia
Source: J Biol Chem. 2021 Sep 25;297(5):101235. doi: 10.1016/j.jbc.2021.101235 (PMC8605249; doi:10.1016/j.jbc.2021.101235)
Supplement: Figures S1 and S2 and Table S1 [file mmc1.pdf]

Supplemental 1

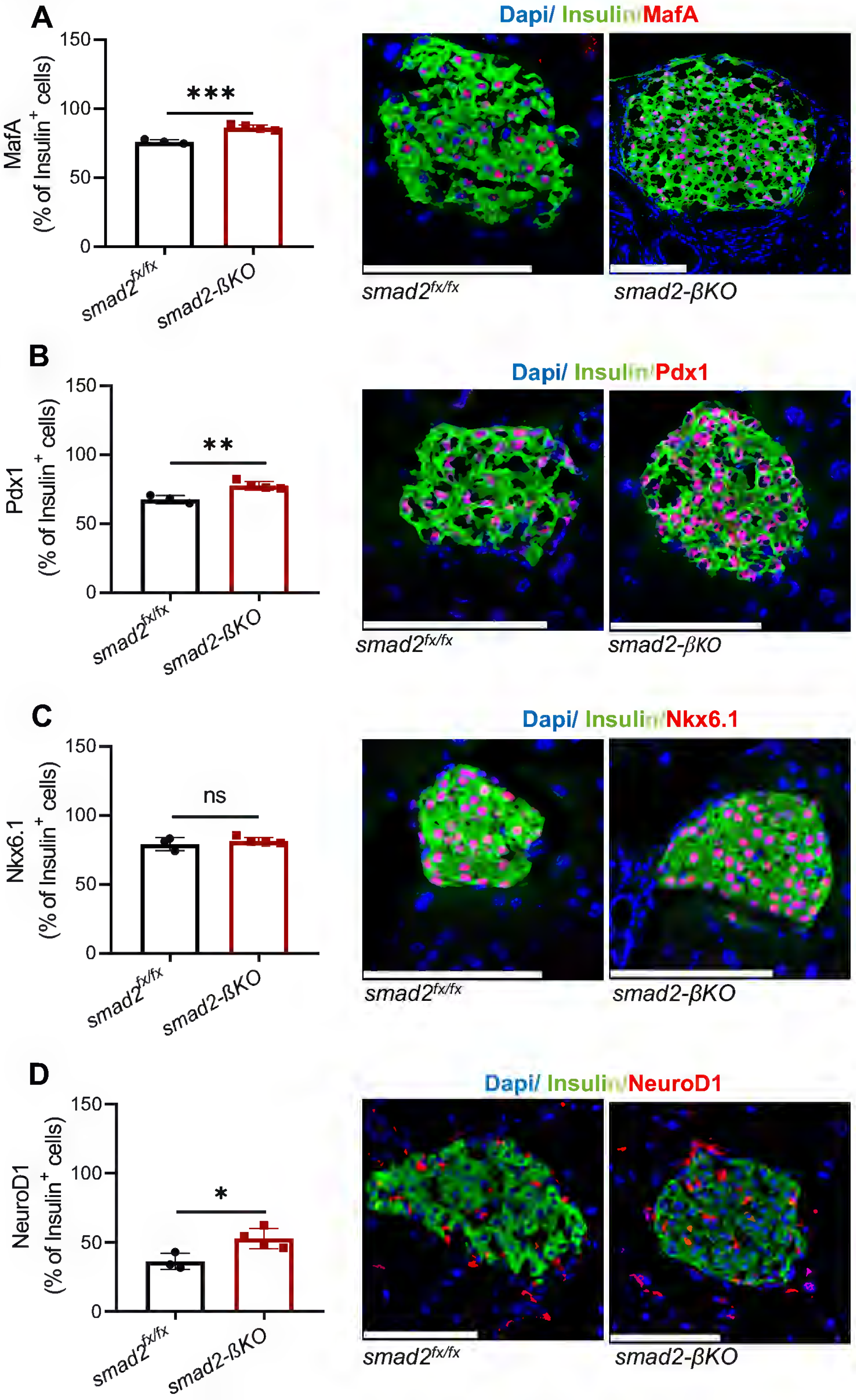

S1. Representative images (right panel) with quantification (left panel) of co-immunostaining for insulin with MafA **(A)**, Pdx1 **(B)**, Nkx6.1 **(C)** and NeuroD1 **(D)** in 14-week-old control (n=3) and smad2-βKO (n=4), showing a higher presence of MafA^{+}, Pdx1^{+}, and NeuroD1^{+} β-cells in smad2-βKO compared to their littermate controls. *$p<0.05$ **$p<0.01$, ***$p<0.001$, ns = no significance. Data are represented as mean ± SD. Scale bar is 100 μm.

Supplemental 2

A

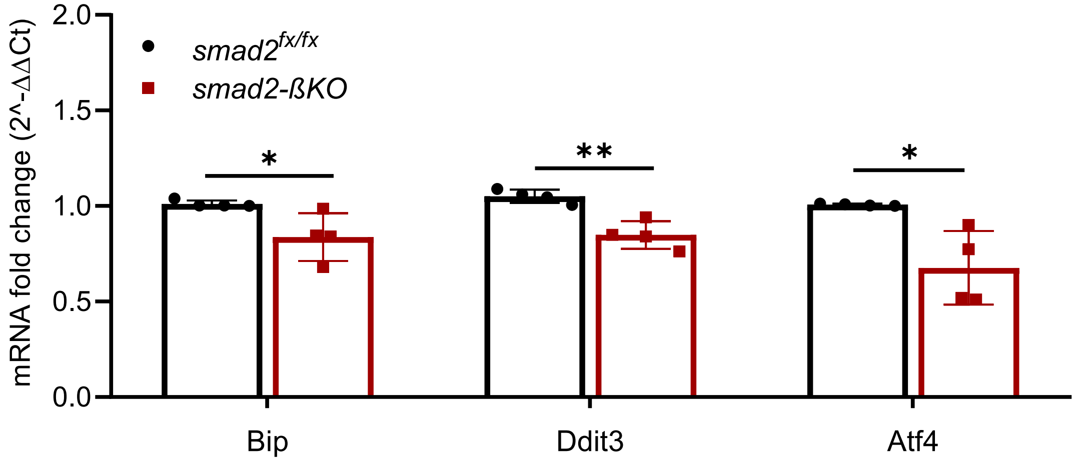

B

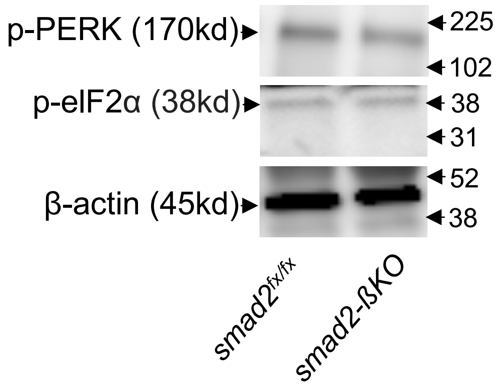

S2. (A) Expression levels of certain ER stress markers (*Bip, Ddit3 and Atf4*) were quantified by RT-PCR in islets isolated from 18-week-old control mice (black circles, n=3) or smad2-βKO mice (red squares, n=4) on regular diet, showing a significantly lower expression of ER stress markers in smad2-βKO islets compared to controls. Values were normalized against the housekeeping gene (*Pipia*), with the latter being consistent across all conditions. **(B)** Islets isolated from control mice or smad2-βKO mice on regular diet, were analyzed for the phosphorylation of URP proteins, p-PERK (Thr980) and p-elF2α (Ser51). Cropped gels are displayed. β-actin was used as a loading and transfer control. Data are represented as mean ± SD. *p<0.05 **p<0.01, ns = no significance.

Supplemental Table 1

List of TaqMan assays used in RT-PCR

Gene	Assay number
Ppia	Mm02342430_g1
Smad2	Mm00487530_m1
Ins1	Mm01259683_g1
Ins2	Mm00731595_gH
Mafa	Mm00845206_s1
Pdx1	Mm00435565_m1
NeuroD1	Mm01280117_m1
NKX6-1	Mm00454961_m1
Pax6	Mm00443081_m1
Cacna1c	Mm01188822_m1
Cacna1d	Mm01209919_m1
Abcc8	Mm00803450_m1
Knj11	Mm0044050_s1
Syt2	Mm00436864_m1
Syt7	Mm00444498_m1
Syt9	Mm00502475_m1
Pclo	Mm00465330_m1
Pick1	Mm00501103_m1
Bip (Hspa5)	Mm00517691_m1
Atf4	Mm00515325_g1
Ddit3 (Chop)	Mm01135937_g1
